# *You talkin’ to me?* Functional breed selection may have fundamentally influenced dogs’ sensitivity to human verbal communicative cues

**DOI:** 10.1186/s12915-024-01983-1

**Published:** 2024-08-26

**Authors:** Petra Dobos, Péter Pongrácz

**Affiliations:** Department of Ethology, Eötvös Loránd University, Pázmány Péter sétány 1/c, Budapest, 1117 Hungary

**Keywords:** Dog, Human, Functional selection, Ostensive cues, Neutral speech, Social learning

## Abstract

**Background:**

The ability to learn from humans via observation was considered to be equally present across properly socialized dogs. We showed recently that cooperative working breeds learned from a human demonstrator more effectively. We hypothesized that functional breed selection could affect sensitivity to human attention-eliciting behavior. Accordingly, we ran the first ever study on dogs that compared the effect of ostensive and neutral verbal communication in a social learning scenario. We used the detour paradigm around a transparent V-shaped fence with either ostensive (addressing the receiver both with words and specific, attention-eliciting prosody) or neutral speech (monotonous reciting of a short poem) demonstration. The other features (gestures, movement) of the demonstration sequence were kept identical between the two conditions. We tested (*N* = 70) companion dogs from 17 cooperative and 16 independent breeds in three 1-min trials. Subjects had to obtain the reward by detouring around the fence.

**Results:**

Detour latencies of the cooperative dogs improved after both ostensive and neutral speech demonstrations. The independent dogs did not improve their detour latency in either of the conditions. Remarkably, ostensive verbal utterances elicited longer relative looking time towards the demonstrator, cooperative dogs looked longer at the demonstrator, and longer looking time resulted in more successful detours.

**Conclusions:**

Our study provides the first indication that functional breed selection had a significant impact on dogs’ sensitivity to ostensive human communication, which, apart from being crucially important for social learning from humans, until now was considered as a uniformly present heritage of domestication in dogs.

**Graphical Abstract:**

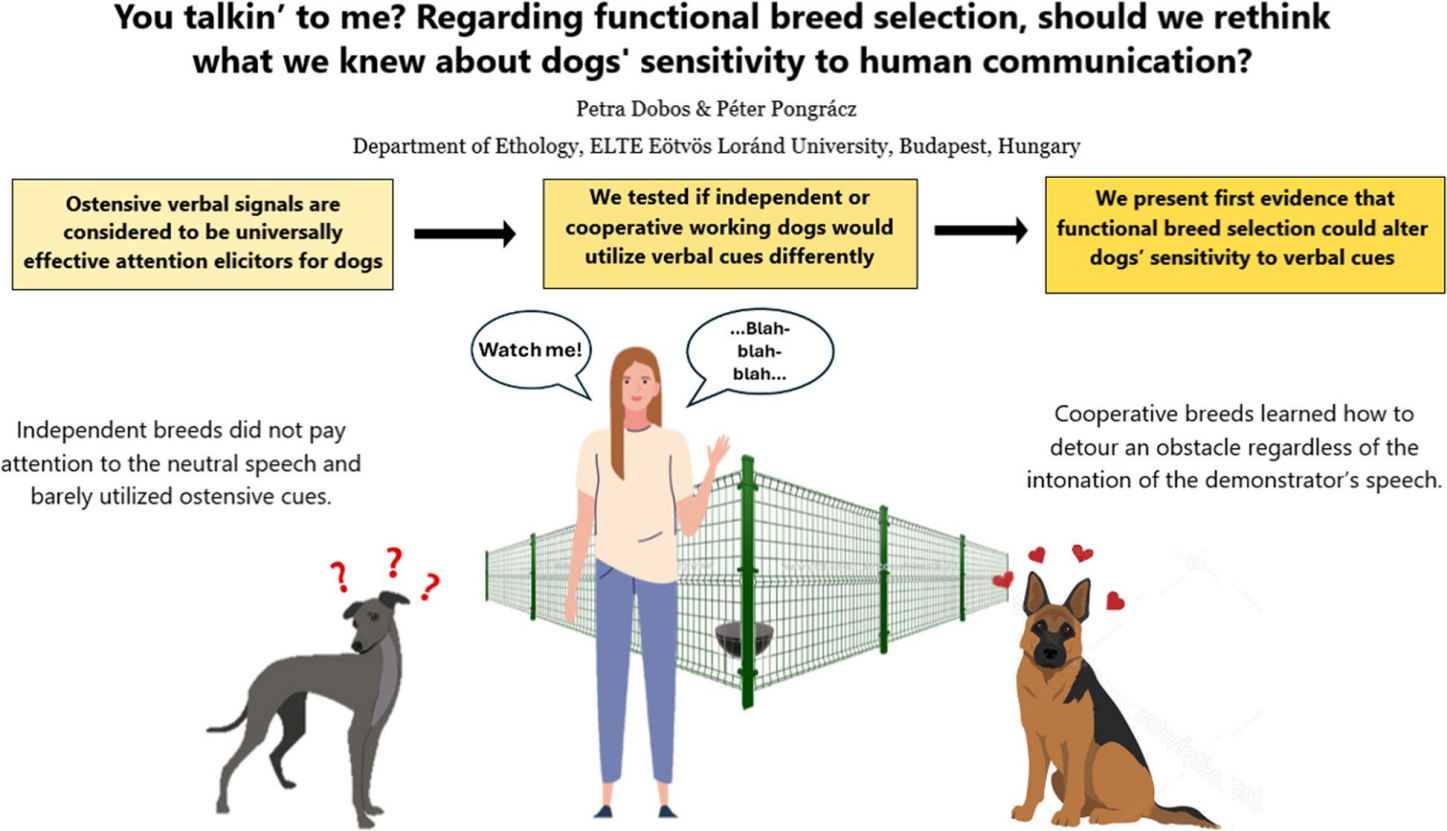

**Supplementary Information:**

The online version contains supplementary material available at 10.1186/s12915-024-01983-1.

## Background

Among terrestrial mammals, there is no match for the dog when it comes to the extent of within-species phenotypic diversity, including the variability of body size [1]. It is readily noticeable regarding the appearance of dogs, for example, there is an approximately 30-fold difference between the adult weight of the smallest and largest dogs (Chihuahua and Great Dane). Dog breeds represent reproductively isolated sub-populations of the species *Canis familiaris*, where reproduction is only allowed between individuals of the same breed, thus relatively high levels of uniformity in the appearance is achieved through artificial selection [2]. Apart from the bewildering array of morphological differences among the various breeds of dogs [3], traditionally they are also characterized by typical behavioral traits that are both mentioned in the official breed standards and utilized in work and sports-related activities [4]. Terms such as “herding dog,” “sighthound” or “sled dog” are telltale signs of the original utilization of the breeds, however, it is commonly assumed that dog breeds would also exhibit different traits of temperament [5] and behavior in everyday contexts such as problem-solving [6] and social interactions with other dogs and humans [7].

Recently, both ethological [8, 9] and molecular genetic investigations have shown [10] that the emergence of dog breeds can have a profound effect on the dogs’ behavioral and socio-cognitive traits (see for a review [11]. By using the responses of more than ten thousand dog owners, Salonen et al. [9] found that dog breed was the strongest factor behind the variability of their seven-trait personality model. Another study [8] utilized almost sixty thousand entries from the Canine Behavioral Assessment and Research Questionnaire (C-BARQ), and by employing latent class analysis they found such clusters that could be useful for characterizing breed-related behavioral/temperament patterns. Since the genetic relationship among the dog breeds, as well as their genetic “distance” from the extant wolves has been established [12], comparing dog breeds based on evolutionary basis became one of the favorite approaches to behavioral breed differences (e.g., [10, 13]). Still, instead of the existence of strictly breed-specific behavioral genotype complexes, molecular geneticists rather argue in favor of more overarching background factors. By comparing over four thousand dogs’ genetic data with behavioral survey results of more than 40 thousand subjects, Dutrow and colleagues [10] described ten canine ancestral lineages and their behavioral correlates among the dog breeds. In another, combined molecular genetics and behavioral study (based on more than 2000 genetic samples and almost 20 thousand surveys), Morrill et al. [14] found no evidence of strictly breed-specific inheritable behavioral factors. However, the strongest behavior-related genetic trait they described was the “biddability-independence” axis, where many dog breeds could be aligned very similarly as the system of functionally selected (working independently vs. cooperatively with humans) breeds would suggest [15].

The distance from the wolf-like ancestor in particular dog breeds seems to be in association with specific behavioral traits that are assumed to belong to the “domestication syndrome” [16]. Based on more than 80 thousand entries from the Swedish Kennel Club’s Dog Mentality Assessment test battery, Hansen-Wheat et al. [17] showed that while “ancient” dog breeds show a consistent negative relationship between their sociability and reactivity indices (as is expected from the domestication syndrome), these traits are more “decoupled” in modern breeds, as evidence of the recent, purpose-specific efforts in selection. However, the clade-based behavioral similarities or differences between the breeds are likely the correlates of genetic relatedness (i.e., the behavior of closely related breeds can be expected to be more similar than compared to a distantly related breed) (e.g., [18]). If one is interested in the effect of more recent events in the evolution of dogs, which happened later than the initial post-domestication divergence of the clades that comprise today’s dog breeds, then it would be useful to find a widely applied force of selection that could have an overarching effect on the behavior of a multitude of breeds. The divergence of “show lines” and “working lines” in particular dog breeds can be mentioned as an example [19], however, it is presently not known whether these changes would be of a similar nature across several breeds of different original functions.

When investigating the effect of artificial selection on the behavioral phenotype of dogs from an ecologically valid point of view, the behavior seen during the various dog–human interactions has fundamental importance. The reason for this is that these behaviors and their corresponding cognitive background enabled dogs to adapt to the anthropogenic niche and become the most successful companion animal for humans. According to the system outlined by Pongrácz and Dobos [20], “true” companion animals are genetically predisposed to develop preferential bonds, and show bi-directional communicative capacity with humans, and once they become socialized, will not leave the company of humans.

Functional working dog selection (i.e., selecting dogs to be efficient in performing a particular task) is one of the promising paradigms, which resulted in two distinct breed categories: cooperative and independently working dogs [21]. Each category contains dozens of dog breeds. They can be characterized with such original tasks that may differ in their nature, but have a common denominator within the given category, either relying on the regular visual and verbal instructions from the handler (cooperative breeds, such as herders, pointers, retrievers); or working on their own, without the aforementioned guidance (independent breeds, such as terriers, sled dogs, scent hounds). It was shown in several studies that independent and cooperative working dogs behaved differently in scenarios that were based on dog–human interactions. When nearly 200 dogs from 56 breeds were tested in a “food stealing” scenario with or without visual access from the owner’s side [22], it was found that the independent working dogs, as well as the without work-function “family style breeds” took the forbidden treat mostly when the owner could not see them. This type of “reward maximizing” with regard to the avoiding of human attention was not found in the cooperative breeds. In the case of visual communicative signals (two-choice human pointing task, with 21 independent and 22 cooperative dog breeds, and a control group of mongrels, 30 dogs in each group, [21]), cooperative dogs performed significantly better than both the independent working breeds and the mongrels. The results were not confounded by the keeping or training conditions of the dogs involved in the study. In a third study, dogs with and without signs of separation-related disorder (SRD), from 8 cooperative and 8 independent breeds were tested in an outdoor separation test [23]. Here, the authors found that cooperative dogs with SRD reacted with more acoustic (barking) and other stress signs (attempts to follow the departing owner) than the independent dogs did, indicating that the cooperative dogs might be more demanding for the presence of their owner. One should note however that although functional selection is mostly considered as a factor above the level of individual dog breeds, recent efforts for selecting purpose-specific working lines within particular breeds could also result in relevant differences in dogs’ human-related behaviors. In the case of Retrievers that were bred for scent-detection work, and were tested in the so-called “unsolvable task,” it was found that those dogs gazed more towards the human handler, who later became successful detection dogs [24].

The behavioral differences found between the two working dog groups can be plausibly explained based on the functional aspect of behavior analysis [25]. For example, when cooperative breeds followed human pointing more successfully [21], this is what we would expect, because cooperative dogs were selected for working in a constant visual feedback loop with their handler. Recently we found that cooperative and independent dogs performed differently in a detour task that involved social learning from a human demonstrator [15]. While cooperative breeds showed significantly improved performance after observing the demonstrator’s action, the independent dogs did not. Again, paying close attention to a human partner would be highly adaptive for the dogs whose original work task required a constant reliance on human signals and commands. Obviously, researchers should always be careful with drawing conclusions based on the functional clustering of dog breeds, because confounders such as the typical keeping conditions [26], or training levels [27] of the cooperative and independent dogs may differ, and this may cause a bias in the performance of the breeds. However, the exact cause (“mechanism”) of functional breed group behavioral difference is still unknown. One could hypothesize that independent and cooperative dogs are not equally sensitive or attentive to such cues that humans provide during their joint actions. For example, it has been shown that dogs rely on human ostensive attention-eliciting cues when learning from a human demonstrator [28]. In that earlier study, when the demonstrator detoured around the obstacle in silence, dogs did not improve the efficiency of their detours, unlike when the demonstrator addressed them with ostensive vocal signals.

Human vocal communication is a rich source of information for dogs. Recently, with state-of-the-art bioacoustics techniques, it was found that dogs show specific sensitivity especially towards particular attention-getting prosody characteristics [29] that humans automatically use when they address dogs (“doggerel” [30]). By using functional magnetic resonance imaging, it was shown that dogs process and react to not only the affective content of verbal utterances (independently of the lexical content), but also to the (already known) words themselves (independently of the intonation) [31]. It was also found that although many dogs can be trained to obey a limited set of verbal commands, a relatively small proportion of dogs have an extraordinary capacity for learning a very high number of words that label objects [32]. Human verbal signals “conveying manifestations of intention to communicate” [33] are called ostensive communication, which has unique lexical (words that address the receiver) and prosody-related characteristics [34]. Ostensive communication is a powerful tool for placing human infants into a “natural pedagogy” context, where consequently the infants can learn effectively and in a somewhat generalized manner from adult instructors [35]. Just as it was seen in human infants, dogs also react to human ostensive cues, but this capacity for showing specific sensitivity to human communicative intent is remarkably missing from intensely socialized (tame) wolves. Becoming sensitive to human ostension is considered to be influenced by the domestication event in the case of the dog [36]. Recently it was also found that even young puppies are sensitive to human visual ostensive signals in a communication context (i.e., indicating the location of a hidden treat with gaze alternation or pointing with an extended arm, but without acoustic attention grabbers, [37]), further strengthening the assumption that this capacity could have been positively selected for in dogs. The sensitivity of dogs to human ostensive signals is considered as an adaptive mechanism that helped effective human–dog coexistence. The fact that cats (a companion animal that retained its independence from humans [38]) did not show comparable sensitivity to human acoustic (lexical and prosody) ostensive signals as the dogs did [39], suggests that in the case of dogs, an asymmetrical, dependence-based relationship with humans, may serve as a basis for their reliance on ostensive signals. According to our recent results, the cooperative dog breeds showed higher performance than the independent working breeds did in a social learning task, where the human demonstrator’s actions were accompanied with acoustic ostensive signals [15]. Hence, we hypothesize that dog breeds that were selected for independent working tasks may lack interest towards human acoustic ostensive cueing, thus they do not pay close attention to the demonstrator during the detour task either. Therefore, in the present investigation, we focused on the potential role of acoustic ostensive signals during the acquisition of a detour task with the help of a human demonstrator in cooperative and independent dogs. In order to test whether the dogs from the two, functionally different working breed types would rely differently on the acoustic ostensive signals, one experimental treatment contained ostensive verbal cueing during the demonstration of the task (addressing the dog both with attention-eliciting words and intonation), while in the other condition, the demonstrator recited a short poem in a neutral (non-ostensive) tone during the detour. The use of neutral speech in this experiment can be considered as an important gap-filler step. To our best knowledge, the specific effect of ostensive acoustic communication on dogs has not been contested yet with the potential effect of neutral verbal utterances in a learning context—which, in turn, would be the most valid control procedure. As ostensive cues can achieve their effect through either (i) signaling the demonstrator’s intent for communication (e.g., [40]), or (ii) simply directing the observer’s attention to the action of the demonstrator (e.g., [41]), we also focused on the potential differentiation between these two mechanisms. We assumed that if dogs would be affected by the attention-enhancing effect of ostension, they would follow more keenly the demonstration accompanied with ostensive signals. However, if ostensive cues rather affect dogs through signifying the demonstrator’s intention to share information with them, we expected that dogs otherwise will follow ostensive and non-ostensive demonstrations with comparable attention.

### Hypotheses

- Question: Did functional breed selection affect the different working breeds’ sensitivity to human ostensive communication?
- Hypothesis A: Cooperative and independent working dog breeds show different sensitivity to human ostensive communication.
- *Prediction A1:* In the case of ostensive speech during demonstration, the relative looking time at the demonstrator will be higher in cooperative dogs’, than in the independent working dogs. No such difference will be seen in the case of neutral speech.
- *Prediction A2:* In the case of ostensive speech during demonstration, the cooperative dogs will detour the fence with higher success rate than in the case of neutral speech. No such difference will be seen in the case of independent working dogs.
- *Prediction A3:* In the case of ostensive speech, cooperative dogs will reach the target significantly faster after the demonstration, than in the case of neutral speech. No such difference will be seen in the case of independent working dogs.
- Hypothesis B: Cooperative and independent working dog breeds show similar sensitivity to human ostensive communication.
- *Prediction B1:* In the case of ostensive speech during demonstration, both cooperative and independent dogs’ relative looking time at the demonstrator will be higher than in the case of neutral speech.
- *Prediction B2:* In the case of ostensive speech during demonstration, both cooperative and independent dogs will detour the fence with a higher success rate than in the case of neutral speech.
- *Prediction B3:* In the case of ostensive speech during demonstration, cooperative dogs will reach the target significantly faster than in the case of neutral speech. No such difference will be seen in the case of independent working dogs (Table 1).

**Table 1:** The hypotheses and predictions in the ostensive and neutral speech conditions in the case of the two breed groups. The predictions where different responses are expected between the two conditions are italicized

	**Ostensive speech demonstration**		**Neutral speech demonstration**	
**Hypothesis**	**cooperative**	**independent**	**cooperative**	**independent**
Breed types show different sensitivity to ostension	High success rate Improving trial-by-trial latency	High * success rate Latency will not improve	*Low success rate* *Latency will not improve*	High * success rate Latency will not improve
Ostension affects the breed types similarly	High success rate Improving trial-by-trial latency	High * success rate Latency will not improve	Low success rate Latency will not improve	*Low success rate* *Latency will not improve*

*Based on the results of Dobos and Pongrácz [15]

## Results

In the case of dogs’ success (i.e., when the dog obtained the reward before a trial ended), we found significant association with the repeated trial factor (Wald *χ*^2^(2) = 15.943; *P* < 0.001); and the keeping conditions of the dog (Wald *χ*^2^(1) = 9.250; *P* = 0.002). Training level (Wald *χ*^2^(1) = 3.171; *P* = 0.075); test group (Wald *χ*^2^(3) = 3.202; *P* = 0.362); and the sex of the dog (Wald *χ*^2^(1) = 2.127; *P* = 0.145) did not have a significant association with dogs’ success. We found no significant interaction between the independent factors. With regard to the significant effects, dogs became more successful in Trial 3 than in Trial 1, and dogs kept indoor only were less successful than dogs who had outdoor access.

**Fig. 1:**
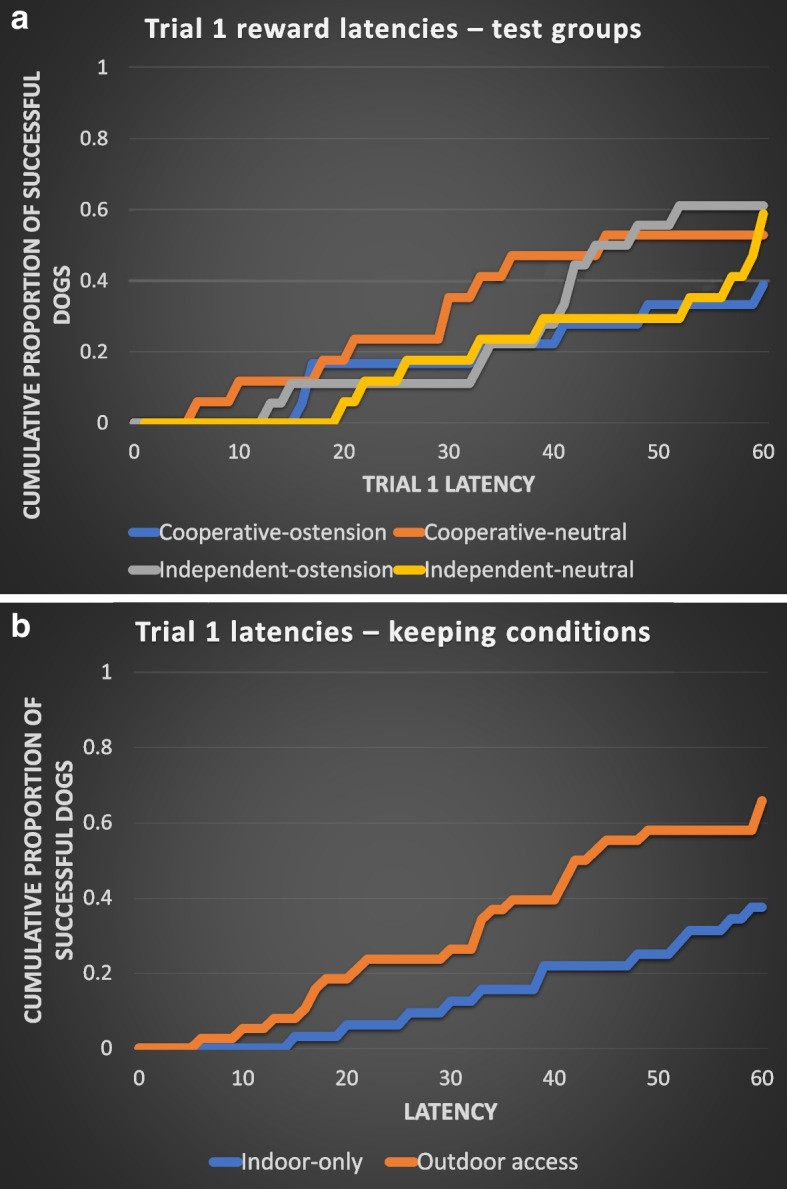
**a** and **b** Cumulative proportion of dogs that performed a successful detour in Trial 1. There was no significant difference between the test groups (**a**). Cooperative breeds-ostensive speech (*N*=18); Cooperative breeds-neutral speech (*N*=17); Independent breeds-ostensive speech (*N*=18); Independent breeds-neutral speech (*N*=17). Indoor-only dogs performed weaker than the dogs who had outdoor access (**b**). Indoor only keeping (*N*=32); Indoor with access to the outdoors keeping (*N*=38)

In the case of the reward latencies, Table 2 shows the results of between-group comparisons. The test group and training level did not have significant associations with the latency in any of the trials. Importantly, dogs in each group performed the detour with similar latencies in Trial 1 (Fig. 1a). Keeping condition had a significant association with the reward latency in Trials 1 and 3. According to this, the dogs that were kept indoors only had significantly weaker performance than the dogs who had access to the outdoors (Fig. 1b).

**Table 2:** Results of the Cox regression analysis in the case of Trial 1, Trial 2, and Trial 3 for the reward latencies. Significant effects are marked with bold letters

**Dependent variable**	**Trial**	**Fixed Factors**	**Chi-square**	**Df**	***p***
Reward latency	1	Groups	1.789	3	0.617
		**Keeping**	**8.577**	**1**	**0.003**
		Training	8.775	5	0.118
	2	Groups	2.993	3	0.393
		Keeping	3.312	1	0.069
		Training	2.479	5	0.780
	3	Groups	6.983	3	0.072
		**Keeping**	**5.100**	**1**	**0.024**
		Training	1.481	5	0.915

We checked whether the distribution of the various keeping conditions was similar across the test groups. GzLM with binary logistics found no significant effect of the test groups on keeping conditions (Wald *χ*^2^(3) = 4.302; *P* = 0.231), which means that the test groups did not differ in their proportions of indoor-only dogs and dogs with outdoor access.

**Fig. 2:**
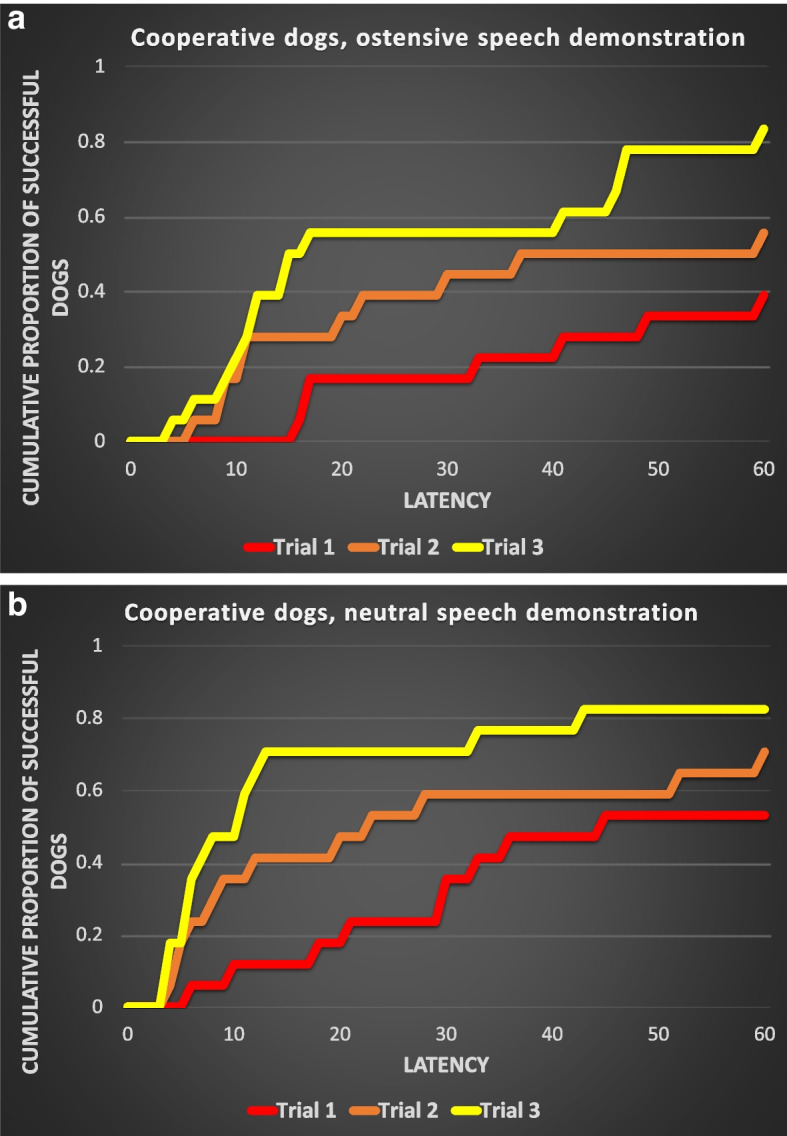
**a** and **b** Cumulative proportions of those cooperative working dogs, who performed a successful detour in the ‘Ostensive speech’ demonstration (**a**, *N*=18) and ‘Neutral speech’ (**b**, *N*=17) demonstration conditions

**Fig. 3:**
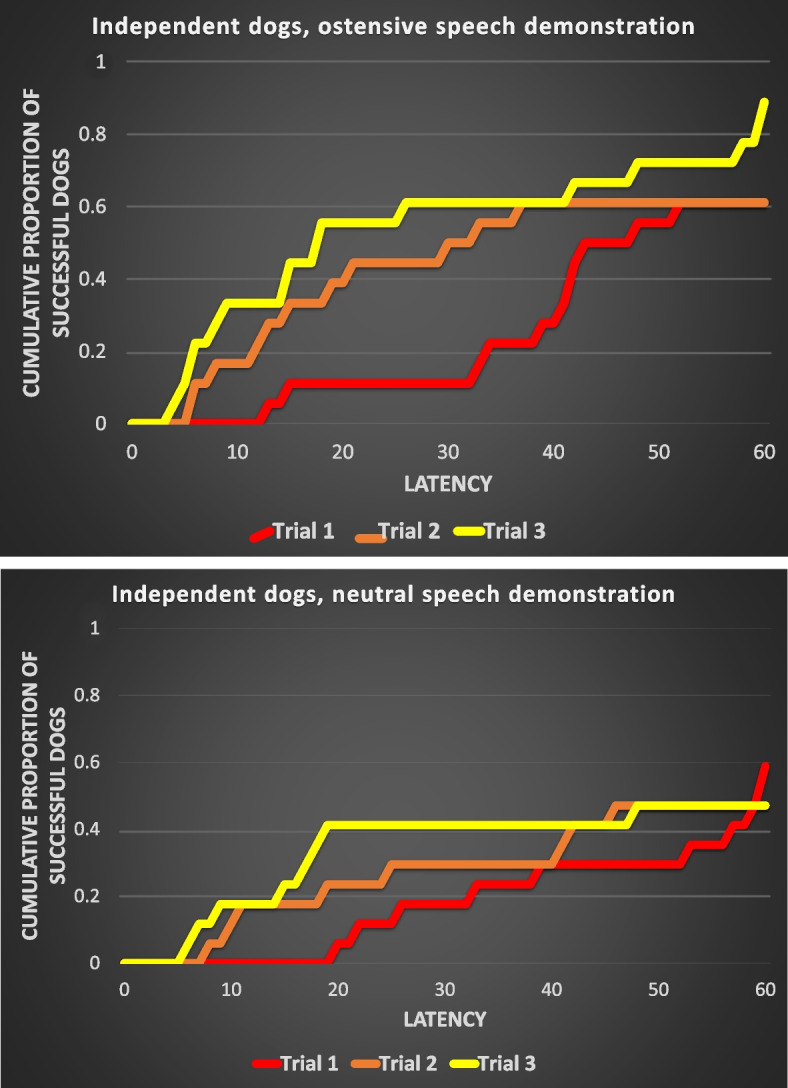
**a** and **b** Cumulative proportions of those independent working dogs, who performed a successful detour in the ‘Ostensive speech’ demonstration (**a**, *N*=18) and ‘Neutral speech’ (**b**, *N*=17) demonstration conditions

Table 3 shows the results of the comparisons of reward latencies across the trials within the test groups. We found a significant effect of the repeated trials in the case of both types of demonstration in the cooperative dog groups (Fig. 2a and b). However, in the case of the independent working dogs, neither the ostensive speech nor the neutral speech demonstration caused a significant change in the reward latencies across the trials (Fig. 3a and b).

**Table 3:** Results of the Cox regression analysis in the case of the two breed groups of dogs and the two test conditions (Ostensive = with ostensive verbal cues during the demonstration; Neutral = with neutral speech “poem” during the demonstration). Significant effects are highlighted with bold letters

**Dependent variable**	**Breed group**	**Test condition**	**Chi-square**	**df**	***p***
Reward (detour) latency	**Cooperative**	**Ostensive**	**9.181**	**2**	**0.010**
		**Neutral**	**6.337**	**2**	**0.042**
	Independent	Ostensive	5.521	2	0.063
		Neutral	0.396	2	0.820

We also analyzed the effect of the two types of verbal utterances (ostensive vs. neutral) during the demonstrator’s action on the reward latencies—separately in the case of the cooperative and independent working dogs. Only Trials 2 and 3 were used, because in Trial 1 there was no demonstration. The type of demonstration had a significant effect on reward latencies in the case of the independent dogs (Wald *χ*^2^(1) = 4.848; *P* = 0.028), here the dogs performed with shorter latencies when they were provided with ostensive speech demonstration. The type of demonstration did not have a significant effect on the reward latencies in the case of the cooperative dogs (Wald *χ*^2^(1) = 2.225; *P* = 0.136), these dogs performed detours with similar efficiency after an ostensive speech and neutral speech demonstration.

**Fig. 4:**
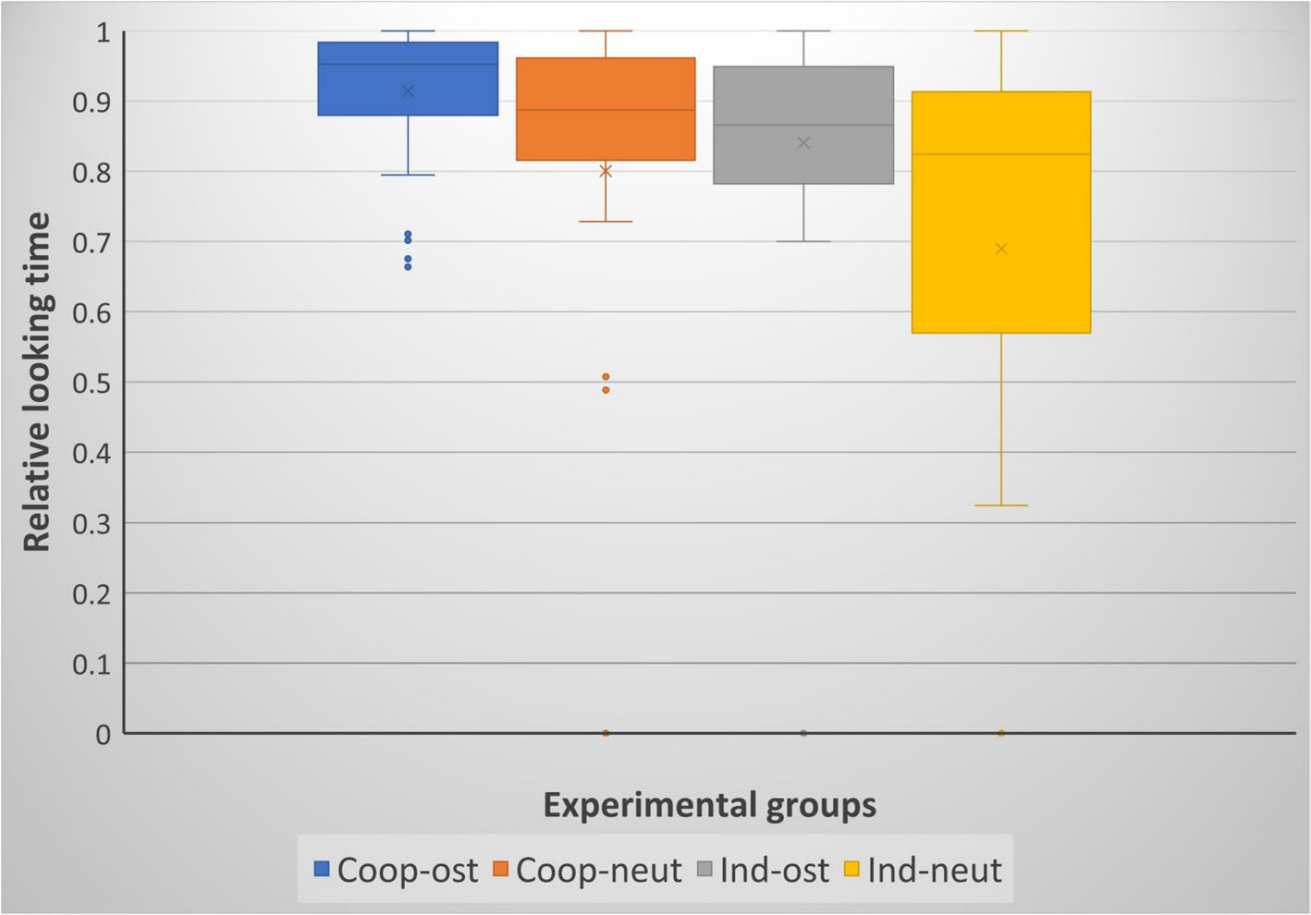
Relative looking time in the case of breed groups and the type of verbal utterances by the demonstrator. Cooperative breeds-ostensive speech (*N* = 36); Cooperative breeds-neutral speech (*N* = 34); Independent breeds-ostensive speech (*N* = 32); Independent breeds-neutral speech (*N* = 34)

In the case of the relative looking time at the demonstrator, we found no significant interactions between the independent variables, however, significant associations were found with the breed group (Wald *χ*^2^(1) = 4.104; *P* = 0.043); demonstration type (Wald *χ*^2^(1) = 8.427; *P* = 0.004); and success (Wald *χ*^2^(1) = 6.578; *P* = 0.010). The repeated trials had no significant effect on the relative looking time (Wald *χ*^2^(1) = 3.511; *P* = 0.061). With regard to the significant effects, cooperative dogs more keenly followed the demonstration, ostensive verbal utterances elicited higher relative looking time (Fig. 4), and longer looking time at the demonstration was more often followed with a successful attempt to detour.

The frequency of looking back at the humans showed a significant association with the trials (*F*_2, 132_ = 3.676; *P* = 0.028), but the test groups had no significant association with it (*F*_3, 66_ = 1.762; *P* = 0.163). With regard to the significant effect, dogs less frequently looked back at the humans in Trial 3 than in the previous trials, which is in agreement with the frequent occurrence of looking back when the task is more difficult for the dogs. The frequency of the owner’s encouraging utterances showed no significant association with the trials (*F*_2, 132_ = 0.328; *P* = 0.721); and we found a weak effect of the test group (*F*_3, 66_ = 3.170; *P* = 0.030). There was no significant difference between the groups according to the post hoc test. We also found no significant effect in the case of the side alternations (trials (*F*_2, 132_ = 2.053; *P* = 0.132); test group (*F*_3, 66_ = 2.295; *P* = 0.086)).

## Discussion

Our research aimed to reveal whether functional breed selection that resulted in cooperative and independent working dogs, affected their responsiveness to human ostensive communication. Furthermore, this was also the first ever study on dogs that compared the effect of ostensive and neutral verbal communication in a social learning scenario. The results confirm our earlier finding [15], namely that cooperative dogs more effectively utilized human demonstration in an observational learning detour task than independent dogs did. However, our new study provided important insight into the mechanism of this difference. Human verbal communication in general, turned to be a more salient attention grabber for cooperative dogs than for independent dog breeds. In contrast to what we expected, cooperative dogs benefitted equally well from ostensive and neutral verbal communication, and they performed the detour faster after observing the demonstrator in both experimental contexts. The independent dogs on the other hand could not improve their performance in either condition. This is a remarkable result because except for a study on cats [39], so far in the perseverative (“A-not-B”) error tests on dogs, the non-ostensive hiding action has never been paired with neutral verbal signals (e.g., like the “poem” condition in our present study). Instead, as a control condition to the ostensive utterances, non-communicative sounds (such as hand clapping, squeaking a toy) were used (e.g., [35, 42]). The same is true for social learning tests, either in manipulation tasks [43], or detour tasks [28], where ostensive verbal cues were contrasted with conditions where the demonstrator acted in silence. Therefore, the previous experiments could not tell whether ostensive verbal signals would be truly necessary for eliciting intense attention from dogs, or simply the verbal activity of the human partner would be a salient enough attention elicitor. From among the potential confounding factors, sex and the training level of the dogs did not affect performance. It was found earlier that female Retrievers relied more on human visual signals than males did [44, 45], but in our study, Retrievers provided only a small proportion of the subjects. Keeping conditions however, affected the success rate of our subjects: indoor-only dogs underperformed those subjects that had access to the outdoors at home. However, our test groups did not differ in the proportion of subjects with different keeping conditions, thus our main results cannot be attributed to the effect of this confounder.

In addition, it has never been tested previously whether the difference in dogs’ attention towards the demonstration would affect their success in social learning (i.e., problem-solving in a detour task). We found earlier that the dogs showed a higher success rate in a search task when they looked at the human demonstrator exactly at the moment of the hiding event [46], and also with the eye-tracking technique, it has been shown that ostensive verbal cueing successfully elicited a gaze following response in dogs [47]. Now we found that dog breeds that were selected for cooperative tasks with their handler have a very strong inclination to pay attention to verbally communicating humans, regardless of the ostensive or neutral nature of their utterances. In contrast to the basic notion so far, namely that domestication resulted in a general and equal sensitivity to human ostensive communication among dogs [36], our results showed that at least in a social learning context, ostension did not surpass the salience of neutral speech as an attention grabber for cooperative dogs. It remains a somewhat intriguing question whether cooperative dogs would learn even from a non-communicating (i.e., silent) demonstrator. Although Pongrácz et al. [28] did not separate the dog breeds into various categories in a study with a similar detour task to the one we used here, they found that a demonstration that was not accompanied with any verbal activity turned out to be ineffective. If we closely examine this earlier study [28], it turns out that in their “silent demonstrator” group, they tested only cooperative dogs (German Shepherd Dog, Belgian Tervueren, Doberman Pinscher, Pointer, and Border Collie) and a single mongrel subject. Therefore, it is likely that even cooperative dogs would not pay intense attention to a demonstrator who detours the fence without a word.

Another novel finding of our study was that ostensive communication elicited higher relative looking time towards the demonstrator’s action, than the neutral intonation speech did. This is in line with the results of a neuroethological fMRI study, which showed that dog brains responded stronger to praising intonation than to neutral speech, even if the speaker was not the dog’s owner [48]. At the same time the fact that the cooperative dogs improved their performance, even with the help of neutral speech-accompanied demonstration, matches to the finding of Andics et al. [31], who described specific activation in the dogs’ brain as a response to the lexical (i.e., intonation-independent) content of meaningful words. While in our test the “poem” recited by the demonstrator did not represent any “meaningful” words for an average dog, we cannot exclude the possibility that cooperative dogs pay elevated attention to every verbal utterance that is provided in an otherwise interesting context for them.

The independent dogs showed differential attention to the demonstrator, depending on her style of verbal communication. Although overall they did not improve even after ostensive cueing, only a non-significant trend was found their detour latencies were shorter in the case of ostensive speech demonstration than in the case of the neutral speech demonstration. This indicates that selection for independent task performance in these breeds probably weakened their general interest for heeding human communication, both in the case of visual [21, 49], and vocal signals. They seemed somewhat interested in the ostensive cues, but they fully neglected the neutral verbal signals. As all the subjects were family dogs, and the groups did not differ in their keeping conditions and levels of training, we can exclude an alternative explanation that the independent and cooperative dogs were exposed to different amounts of verbal communication from their owners. Our results therefore convincingly support the assumption that functional breed selection affected dogs’ attentiveness towards human communication. This in turn can affect their behavior in such interactive activities such as social learning and other tasks that require synchronization with humans.

Our results also suggest that during the solution of the detour task, the underlying mechanism of social learning for the dogs could likely be response facilitation. Response facilitation signifies that the observation of a demonstrated behavior elicits a similar behavioral response in the observer, when this particular behavior has already been in the repertoire of the observer [50]. This notion can be corroborated by the facts that (i) detouring is likely included in the behavioral repertoire of dogs; (ii) dogs with access to the outdoors performed better; and (iii) in the case of local/stimulus enhancement (i.e., another likely social learning mechanism in the case of learning the detour), the longer time spent with watching the demonstration would likely not lead to higher success. In this latter case, for a more successful detour, it would be enough if the dogs would only pay attention to the demonstrator as she detours the very end of the fence [46]. The outdoor dogs’ success can be related to the fact that they could encounter more fences and naturally occurring “detour tasks” during their lifetime, and thus, this task can be more relevant and/or familiar for them. An alternative explanation could be that indoor dogs can only go out when their owners take them for walks, and as the free off-leash opportunities are becoming increasingly rare because of legal regulations, these dogs have much more limited experience with “spatial problem-solving” than those canines that can freely enjoy the outdoors on a regular basis. Regarding any of the aforementioned reasons, conceivably, indoor-outdoor dogs can better understand the concept of a fence, or they are more used to the action of running along one. However, the experimental groups were equalized for the distribution of various keeping conditions, therefore our main finding could not be explained by this difference.

## Limitations to the study

We found a clear difference between the social learning performance of independent and cooperative working dogs as a function of human acoustic signaling during the demonstration phase of the trials. However, the mechanism and functional causation behind the surprising efficacy of cooperative dogs in utilizing even the neutral speech demonstration scenario has so far remained undiscovered. We suggest at least two concurrent explanations: (1) the “*communication-based*”* theory* would suggest that in the cooperative dogs, there was a selection for higher sensitivity to the communicative intent of humans, therefore human verbal signals for them would be salient enough even with a neutral prosody/intonation. (2) On the other hand, the “*higher sensitivity toward humans*”* theory* would assume that the presence (visual, olfactory, acoustic cues) of a human demonstrator puts the cooperative dogs into an attentive state, enabling them to learn better from the human’s behavior. It is an interesting opportunity for future experiments to disentangle the two explanations from each other.

A further limitation for our study was that we relied on the traditional dog breed descriptions, when we selected the candidates for the independent and cooperative working dog groups. As it was shown in the case of the Kelpie, their divergence from the still primarily livestock herder Australian Working Kelpie resulted in heritable differences between the two types, affecting mainly the chromosomal regions responsible for pain-resilience and fear-related memory formation [51]. Thus, when we sort the dog breeds to groups according to their historical function, there is a chance that the individual dogs taken to our tests by the owners have already departed somewhat from the original behavioral characteristics.

A related issue could emerge from the complex ancestral background of such modern dog breeds that were “created” from different landraces and older dog breeds, often with various functional predispositions [12]. Again, we should be cautious when deciding where to place these dog breeds along the independence-cooperativity axis.

Finally, the apparently smaller average body size of the independent dogs could result in slower detours, given that the smaller dogs have to cover a relatively longer route to the reward. However, the test groups themselves did not have a significant effect neither on the success rate nor the detour latencies of the dogs.

## Conclusions

Our results confirm that functional breed selection affected dogs’ attentiveness towards human communication. Our experiment was based on an ecologically valid context, where the independent and cooperative dogs were placed into a scenario that tapped their social interactivity with humans in a biologically meaningful way.

So far, the main approach stated that domestication caused sensitivity to human ostensive communication uniformly among dogs [33, 36, 37]. However, domestication and breed selection have an interdependent relationship, therefore looking through the ecologically valid filter of functional breed selection, we can notice interesting nuances of these often-generalized socio-cognitive features in dogs.

For instance, we could now show that ostension is not omnipotent over neutral speech if the dog has been selected for cooperativity and attentiveness. At the same time, among independent breeds, we found indirect evidence that their interest in humans, and their sensitivity to human communication, could become attenuated while their independent resourcefulness was enhanced during functional selection.

## Methods

### Subjects

We tested adult companion dogs *N* = 70 (minimum 1 year, maximum 12 years old, mean age ± SD = 4.2 ± 3.1 years) independently from their sex or reproductive status. Dog owners were recruited through advertisements on social media. We specified which dog breeds we were looking for (visually cooperative or visually independent working breeds), and we also required that the subjects had not previously participated in a detour test. Experimental groups were assembled in a parallel manner, and dogs were assigned to the ostensive and neutral speech groups randomly, with extra attention given that none of the breeds were overrepresented in any of the groups.

Table 4 shows the basic demographic details of the subjects (breed, breed group, age, and sex) as well as their genetic clade assignments. We took special care to invite representatives of both breed groups (independent and cooperative) from the widest possible range of breeds. Eventually, we tested 17 breeds from the cooperative working group and 16 breeds from the independent working group. We also recorded the keeping conditions of the subjects (indoor only, indoor–outdoor, and outdoor only), as well as the level of training the dogs had received (none, training at home, course at dog school, regular dog school, assigned trainer, and specific sports/work training).

**Table 4:** List of the participating dogs whose data were included to the statistical analyses. We indicate the test group assignments, where Coop-o = cooperative dogs with ostensive speech demonstration; Coop-n = cooperative dogs with neutral speech demonstration; Ind-o = independent dogs with ostensive speech demonstration; Ind-n = independent dogs with neutral speech demonstration

	**Test group**	**Breed**	**Breed type**	**Age**	**Sex**	**Clade**
1	Coop-o	Border Collie	Cooperative	1.5	Male	T
2	Coop-o	Lagotto Romagnolo	Cooperative	3	Male	R^a^
3	Coop-o	Australian Shepherd	Cooperative	4	Male	T
4	Coop-o	Labrador Retriever	Cooperative	2	Female	Q
5	Coop-o	Border Collie	Cooperative	2	Male	T
6	Coop-o	Lagotto Romagnolo	Cooperative	4	Female	R^a^
7	Coop-o	Border Collie	Cooperative	7	Male	T
8	Coop-o	Pumi	Cooperative	4	Male	G
9	Coop-o	Briard	Cooperative	1	Male	S
10	Coop-o	German Shepherd Dog	Cooperative	10	Male	M
11	Coop-o	Shetland Sheepdog	Cooperative	3	Male	T
12	Coop-o	Mudi	Cooperative	1	Male	G^b^
13	Coop-o	Labrador Retriever	Cooperative	2	Male	Q
14	Coop-o	Bouvier des Flandres	Cooperative	1	Female	S
15	Coop-o	Pumi	Cooperative	4	Male	G
16	Coop-o	Rough Collie	Cooperative	11	Female	T
17	Coop-o	Vizsla	Cooperative	8	Male	R
18	Coop-o	Australian Shepherd	Cooperative	2.5	Female	T
19	Coop-n	Border Collie	Cooperative	2.5	Female	T
20	Coop-n	Australian Shepherd	Cooperative	3	Male	T
21	Coop-n	Irish Setter	Cooperative	5.5	Female	R
22	Coop-n	Golden Retriever	Cooperative	3	Male	Q
23	Coop-n	Border Collie	Cooperative	1	Male	T
24	Coop-n	Irish Setter	Cooperative	11	Male	R
25	Coop-n	Pointer	Cooperative	8	Female	R
26	Coop-n	Rottweiler	Cooperative	8.5	Male	U
27	Coop-n	Border Collie	Cooperative	6	Female	T
28	Coop-n	Rottweiler	Cooperative	5.5	Female	U
29	Coop-n	Irish Setter	Cooperative	1.5	Male	R
30	Coop-n	Border Collie	Cooperative	2.5	Female	T
31	Coop-n	Border Collie	Cooperative	3	Female	T
32	Coop-n	Puli	Cooperative	9	Female	G
33	Coop-n	Puli	Cooperative	9	Female	G
34	Coop-n	German Shepherd Dog	Cooperative	8	Female	M
35	Coop-n	Labrador Retriever	Cooperative	6	Male	Q
36	Ind-o	Dachshund	Independent	1	Female	O
37	Ind-o	Dachshund	Independent	4	Female	O
38	Ind-o	Dachshund	Independent	5	Male	O
39	Ind-o	Dachshund	Independent	7	Female	O
40	Ind-o	Transylvanian Hound	Independent	5	Male	n/a
41	Ind-o	Fox Terrier	Independent	5	Female	L
42	Ind-o	Borzoi	Independent	2	Male	T
43	Ind-o	Fox Terrier	Independent	2.5	Female	L
44	Ind-o	Jack Russell Terrier	Independent	3.5	Male	L
45	Ind-o	Bedlington Terrier	Independent	2	Female	L
46	Ind-o	Bull Terrier	Independent	10.5	Female	W
47	Ind-o	Akita Inu	Independent	2	Female	A
48	Ind-o	Hovawart	Independent	4	Male	n/a
49	Ind-o	Hovawart	Independent	12	Female	n/a
50	Ind-o	Transylvanian Hound	Independent	1	Male	n/a
51	Ind-o	Transylvanian Hound	Independent	3	Female	n/a
52	Ind-o	Yakutian Laika	Independent	2	Male	A^c^
53	Ind-o	Yakutian Laika	Independent	1	Male	A^c^
54	Ind-n	Dachshund	Independent	11	Female	O
55	Ind-n	Dachshund	Independent	7	Female	O
56	Ind-n	Chinese Shar Pei	Independent	2.5	Female	A
57	Ind-n	Dachshund	Independent	11	Female	O
58	Ind-n	Jack Russell Terrier	Independent	4.5	Male	L
59	Ind-n	Greyhound	Independent	2.5	Male	T
60	Ind-n	Basset Hound	Independent	2.5	Female	O
61	Ind-n	Jack Russell Terrier	Independent	3	Female	L
62	Ind-n	Whippet	Independent	5	Female	T
63	Ind-n	Whippet	Independent	1	Male	T
64	Ind-n	Whippet	Independent	1.5	Female	T
65	Ind-n	Whippet	Independent	2	Male	T
66	Ind-n	Jack Russell Terrier	Independent	7	Male	L
67	Ind-n	Samoyed	Independent	4	Female	B
68	Ind-n	Shiba Inu	Independent	7	Male	A
69	Ind-n	Shiba Inu	Independent	4	Female	A
70	Ind-n	Samoyed	Independent	1	Male	B

Genetic clade assignments of the dog breeds were mostly based on [12], with exception of ^a^ = [52]; ^b^ = [53], ^c^ = https://embarkvet.com/resources/dog-breeds/yakutian-laika/ (accessed on February 25, 2024); n/a = no available data for genetic clade assignment

### Equipment

The experiment was conducted outdoors in the park area of Eötvös Loránd University, Budapest, Hungary. We performed all tests between October 2023 and December 2023. Our main equipment was a V-shaped, transparent wire-mesh fence, with 3 m long, 1 m high wings. The intersecting angle at the corner of the fence was set to 80 degrees. With protruding steel pegs, the fence was firmly inserted into the ground so that its lower edge was just above the soil, preventing the dogs from digging or going under the fence.

**Fig. 5:**
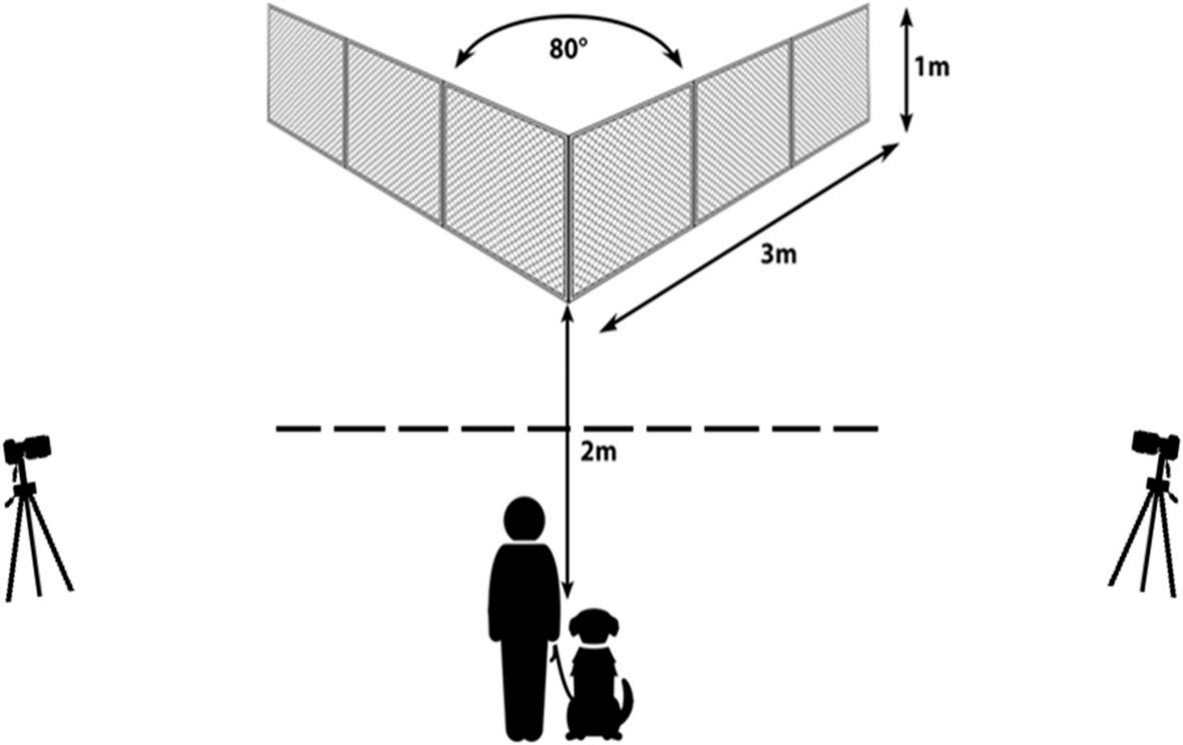
The experimental setup, based on Dobos and Pongrácz [15]. The owner and the dog stand at the starting point. The reward was always placed to the inside corner of the fence about 20 cm away from the corner

A starting point was marked 2 m away from the corner of the V-shaped fence in the midline. We recorded the tests with two video cameras (one Blow GoPro and one Panasonic) that were positioned on tripods and placed to the left and right of the V-shaped fence approximately in line with the front corner. The outlay of the testing area with the V-shaped fence can be seen in Fig. 5.

### Experimental groups

Each subject was tested once, in only one experimental group (Table 5). We assigned the dogs to the experimental groups with special attention given to the balanced distribution of sex, age, keeping condition, and training level of the subjects.

**Table 5:** The experimental groups

**Treatment**	**Trials**	**Independent working dogs**	**Cooperative working dogs**
Ostensive speech	1. No demonstration 2. Demonstration 3. Demonstration	*N* = 18 individuals	*N* = 18 individuals
Neutral speech	1. No demonstration 2. Demonstration 3. Demonstration	*N* = 17 individuals	*N* = 17 individuals

### Procedure

Upon their arrival, the experimenter (E) explained the test procedure to the owner (O), who gave their written informed consent that they were told about the circumstances and general aims of the study. We asked the O whether the dog was motivated best with food or toy, and we used the reward selected by the O. At first, the O was allowed to walk the dog on a leash around the testing area, but we asked them to not let the dog approach the experimental fence yet. Then we asked the O to position the dog on the starting point, facing towards the fence. At that point, the dog was still on leash.

In the first trial, the E called the dog’s attention (by calling its name and saying, for example, “Look”). Then, the E turned her back to the dog and walked to the intersecting angle of the fence, visibly holding a piece of food (or the toy) in her hand, leaned over the fence, and dropped the reward to the inner corner of the fence. After this, the E showed her empty hands to the dog. Then, she returned to the starting point and stood next to the O. At that moment the O was requested to release the dog. The O was instructed to encourage the dog to obtain the reward. We requested the O’s not to use commands such as “Forward”, “Go around” or “Go further”. Gestural commands were also disallowed. During this study, we did not have to exclude any subjects because the owner broke the verbal command rules.

The dog had 60 s to solve the task. If it performed a successful detour within the time limit and obtained the reward from behind the fence, the O had to recall the dog to the starting point, and the next trial started. If the dog did not perform a successful detour in 60 s, the trial ended, and the O had to position the dog back to the starting point. Two consecutive trials were separated by approximately 1 min inter-trial intervals.

#### Ostensive speech demonstration groups

In this group before Trial 2 and Trial 3, the E demonstrated a detour to the dog. During the demonstration, the O had to keep the dog on a leash at the starting point. The E held the reward in her hand, stepped in front of the dog, then turned her back towards the dog and started to walk along one wing of the V-shaped fence. While performing the demonstration, E kept calling the dog’s attention with ostensive speech (telling the dog’s name, repeating words such as “Look,” “Here I go,” etc.). First, the E walked along the outside of the fence wing, then turned in at the end, and came back along the inner side of the wing towards the corner. When she arrived at the inner corner, she held up the reward for a moment, and then put it down on the plate. Then she showed her empty hands towards the dog and walked out along the other wing of the fence, still keeping the dog’s attention on herself with the usual ostensive utterances. When E returned to the starting point, the dog was released and encouraged to get to the reward.

In Trial 3, the demonstration was identical to the one described in the case of Trial 2 with the exception that E performed the detour from the opposite direction (i.e., if Trial 2 had a left-to-right detour direction, in Trial 3 E walked right-to-left).

The direction of the detour demonstration in Trial 2 was also based on the direction of the dog’s successful detour in Trial 1: the E always started the demonstration on the opposite side of the fence than the dog used. In case of an unsuccessful Trial 1 (i.e., the dog could not detour the fence within 1 min), E randomly chose the side in Trial 2 for the demonstration.

#### Neutral speech demonstration groups

The trials were the same, and the E performed the same actions and movements as described in the *Ostensive demonstration group*, except that in the demonstration trials (Trial 2 and 3) the E instead of making the detour with ostensive communication (calling the dog’s name, etc.) repeated a short poem (in Hungarian) in a neutral tone.

### Exclusions

We excluded subjects that were not motivated to perform any trials or lost interest for further performance during the test. A dog was considered to have lost interest if it did not approach the V-shaped fence upon its release from the starting point or only approached it once. We had to exclude *N* = 6 dogs altogether for this reason. Their distribution in the four test groups was as follows: cooperative/neutral speech demo *N* = 1; independent/neutral speech demo *N* = 5. These non-motivated dogs had the following training backgrounds: nothing *N* = 2; course at dog school *N* = 3 and specific work/sports training *N* = 1.

We had to exclude an additional dog because it accidentally saw another dog’s detour; cooperative/ostensive speech demo *N* = 1. Another dog was excluded because it was released by its owner during demonstration; cooperative/neutral speech demo *N* = 1. The results of the excluded dogs did not appear in the statistical analysis.

### Behavioral coding

The tests were recorded with two cameras. We used BORIS software (© Olivier Friard and Marco Gamba, [54]) for the extraction of data from the video sequences. The examined variables used for the analysis are shown in Table 6. For the inter-coder reliability analysis, 20% of the videos were re-coded by a second experimenter who was unaware of the breed group assignment of the subjects and the experimental hypotheses.

**Table 6:** The list and description of behavioral variables used in this study

**Behavioral variable**	**Unit**	**Description**
Success	Occurrence (0–1)	The dog reaches the reward after performing a successful detour, and then it touches/consumes the reward
Reward latency	(s)	The time elapsed between the moment of releasing the dog by the owner at the starting point and the dog’s arrival to the reward (i.e., after a successful detour). In the case of an unsuccessful trial, *60 s* was assigned
Looking back	1/s	During attempts to detour, the dog turns towards the owner/experimenter (by turning its head only, or with full body orientation) and looks at them. The *number of looking back* events is then divided by the *reward latency*
Side alternation (at corner)	1/s	The *number of swapping the side* events at the corner of the fence during the dog’s attempts to detour, divided by the *reward latency*
Encouragement (by owner)	1/s	The *number of distinct verbal utterances* (at least 1 s between two adjacent ones) given by the owner during the dog’s attempts to detour, divided by the *reward latency*
Demonstration duration	(s)	The duration of the Experimenter’s demonstration, measured between her departure from the starting point and arrival back to the external apex of the fence
Demonstration looking time	Time percentage %	During demonstration, the dog looks at the demonstrator. The *overall duration of looking* is then divided by the *duration of the demonstration*

## Statistical analysis

All statistical analyses were performed with the SPSS.22 software. Whenever possible, the biologically meaningful 2-way interactions were included to the models. We applied backward model selection until only the main effects and significant interaction(s) remained in the model. We report the final (simplest) model in each instance.

The success rate of dogs was analyzed with Generalized Estimating Equations (GEE) with binary logistics. We added the test group as an independent factor and the dog’s sex, reproductive status, keeping condition, and training level as covariates. The trial served as a repeated factor. The 2-way interactions between the test group and the other factors were also included. Regarding keeping conditions, we had only two outdoor-only dogs, these we clustered to the indoor-outdoor dogs.

Frequencies (looking back, side alternation, encouragement) were analyzed with GLM with trial used as a repeated factor. The testing group was used as an independent factor.

Reward latencies were analyzed with Cox regression models. We performed three types of comparisons. First, we compared the latencies of the trials separately between testing groups. To these models, keeping conditions and training levels were also added as independent factors. The main goal here was to see whether dogs in the different experimental groups showed different performance in Trial 1 (without demonstration), which would indicate an initial difference of their ability to detour the fence. Second, we compared the reward latencies between trials, within the experimental groups to see whether dogs improved their detour speed (i.e., did they learn compared to their performance in Trial 1). Third, we analyzed the effect of the type of verbal utterances (ostensive vs. neutral speech) of the demonstrator. Here we compared the latencies of Trials 2–3 between the ostensive and neutral speech demonstration groups, separately in the case of cooperative and independent breed groups.

We also analyzed the relative looking time the dogs spent watching the demonstrator’s action. This parameter was calculated by dividing the total time of watching the demonstrator with the duration of demonstration. We used GEE with a linear function, where trials (2–3) served as a repeated factor, breed type (independent vs. cooperative) demonstration (ostensive vs. neutral speech), and success (1 vs. 0) were used as independent variables. Two-way interactions have been added to the model as well.

To check the reliability of the coding method, an independent observer (who was blind to the test hypotheses) coded video footage from 13 randomly chosen dogs. Based on the analysis, our coding procedure was reliable (Spearman’s rho—reward latency: *R*_(39)_ = 0.998; *p* < 0.001; “Encouragement” frequency: *R*_(39)_ = 0.568; *p* < 0.001; “Looking back” frequency: *R*_(39)_ = 0.956; *p* < 0.001; “Side alternation” frequency: *R*_(39)_ = 0.974; *p* < 0.001; demonstration looking time: *R*_(26)_ = 0.920; *p* < 0.001).

### Supplementary Information

Supplementary Material 1

## Data Availability

A video abstract, showing the experimental procedure, can be found at: https://doi.org/10.6084/m9.figshare.26662573.v1. All data generated or analyzed during this study are included in this published article [and its supplementary information file Additional_file1.csv].

## Abbreviations

Coop-o: Cooperative dogs with ostensive speech demonstration
Coop-n: Cooperative dogs with neutral speech demonstration
Ind-o: Independent dogs with ostensive speech demonstration
Ind-n: Independent dogs with neutral speech demonstration
E: Experimenter
O: Owner (of the dog)
